# Involvement of the eIF2α Kinase GCN2 in UV-B Responses

**DOI:** 10.3389/fpls.2019.01492

**Published:** 2019-11-28

**Authors:** Paula Llabata, Julia Richter, Isabel Faus, Karolina Słomiňska-Durdasiak, Lukas Hubert Zeh, Jose Gadea, Marie-Theres Hauser

**Affiliations:** ^1^Instituto de Biología Molecular y Celular de Plantas (IBMCP), Universidad Politécnica de Valencia (UPV), Consejo Superior de Investigaciones Científicas (CSIC), Ciudad Politécnica de la Innovación (CPI), Valencia, Spain; ^2^Institute of Applied Genetics and Cell Biology, BOKU University of Natural Resources and Life Sciences, Vienna, Austria; ^3^Bellvitge Biomedical Research Institute IDIBELL, Barcelona, Spain; ^4^Leibniz Institute of Plant Genetics and Crop Plant Research (IPK), Gatersleben, Germany

**Keywords:** protein synthesis, abiotic/environmental stress, cell signalling, gene expression, post-translational regulation, DNA damage, puromycin

## Abstract

*GCN2* (*general control nonrepressed 2*) is a serine/threonine-protein kinase that regulates translation in response to stressors such as amino acid and purin deprivation, cold shock, wounding, cadmium, and UV-C exposure. Activated *GCN2* phosphorylates the α-subunit of the eukaryotic initiation factor 2 (eIF2) leading to a drastic inhibition of protein synthesis and shifting translation to specific mRNAs. To investigate the role of *GCN2* in responses to UV-B radiation its activity was analyzed through eIF2α phosphorylation assays in mutants of the specific UV-B and stress signaling pathways of *Arabidopsis thaliana*. EIF2α phosphorylation was detectable 30 min after UV-B exposure, independent of the UV-B photoreceptor *UV RESISTANCE LOCUS8* and its downstream signaling components. GCN2 dependent phosphorylation of eIF2α was also detectable in mutants of the stress related MAP kinases, *MPK3* and *MPK6* and their negative regulator *map kinase phosphatase1* (*MKP*1). Transcription of downstream components of the UV-B signaling pathway, the *Chalcone synthase* (*CHS*) was constitutively higher in *gcn2-1* compared to wildtype and further increased upon UV-B while *GLUTATHIONE PEROXIDASE7* (*GPX7*) behaved similarly to wildtype. The UVR8 independent *FAD-LINKED OXIDOREDUCTASE* (*FADox*) had a lower basal expression in *gcn2-1* which was increased upon UV-B. Since high fluence rates of UV-B induce DNA damage the expression of the *RAS ASSOCIATED WITH DIABETES PROTEIN51* (*RAD51*) was quantified before and after UV-B. While the basal expression was similar to wildtype it was significantly less induced upon UV-B in the *gcn2-1* mutant. This expression pattern correlates with the finding that *gcn2* mutants develop less cyclobutane pyrimidine dimers after UV-B exposure. Quantification of translation with the puromycination assay revealed that *gcn2* mutants have an increased rate of translation which was also higher upon UV-B. Growth of *gcn2* mutants to chronic UV-B exposure supports *GCN2*’s role as a negative regulator of UV-B responses. The elevated resistance of *gcn2* mutants towards repeated UV-B exposure points to a critical role of *GCN2* in the regulation of translation upon UV-B.

## Introduction

UV-B (280–315 nm) is the most harmful radiation of the sun’s spectrum reaching the biosphere. Thanks to the stratospheric ozone layer the extremely damaging solar UV-C (100–280 nm) is completely absorbed, while about 10% of the UV-B reaches the Earth’s surface. Thus plants are naturally never exposed to UV-C but to high-energy UV-B wavelengths mainly above 295 nm. High levels of UV-B damages RNA, DNA and represses its replication, impairs translation and proteins, triggers reactive oxygen species, and lead to severe growth retardation in maize and *Arabidopsis* (Jansen et al., 1998; Britt, 2004; Casati and Walbot, 2004a; Qüesta et al., 2013; Lario et al., 2015). However, low levels of UV-B serve as signal for development such as photomorphogenesis and inhibition of hypocotyl elongation. UV-B stimulates the synthesis of UV-B and reactive oxygen species scavenging secondary metabolites of the phenylpropanoid pathway, for instance flavonoids and anthocyanins (Tilbrook et al., 2013; Jenkins, 2017; Liang et al., 2019). The nucleocytoplasmic *UV RESISTANCE LOCUS8* (*UVR8*) is sensing UV-B (Rizzini et al., 2011; Christie et al., 2012; Wu et al., 2012). In the absence of UV-B UVR8 forms homodimers in the cytoplasm which dissociate upon photoreception. Monomeric UVR8 interacts with a key regulator of photomorphogenesis, the E3 ubiquitin ligase *CONSTITUTIVELY PHOTOMORPHOGENIC1* (*COP1*). This interaction is essential for UVR8 accumulation in the nucleus (Oravecz et al., 2006; Favory et al., 2009). The UVR8/COP1 interaction is also crucial for the expression and stability of the transcription factors *ELONGATED HYPOCOTYL5* (*HY5*) and its homolog *HYH* (Ulm et al., 2004; Stracke et al., 2010; Rizzini et al., 2011; Huang et al., 2013; Binkert et al., 2014). Brown and Jenkins (2008) found that UVR8 dependent and independent genes exhibit different needs for fluence rates. The UVR8-COP1-HY5/HYH specific pathway activates genes below 1 µmol m^-2^ s^-1^ or even lower (0.1 µmol m^-2^ s^-1^) while the independent genes were stimulated above 1 µmol m^-2^ s^-1^ UV-B. Among low fluence rate UVR8 dependent genes are HY5, HYH, and their downstream targets CHALCONE SYNTHASE (CHS) and *GLUTATHIONE PEROXIDASE7 (GPX7)*. Among the UV-B induced but UVR8 independent genes are for example *FAD-LINKED OXIDOREDUCTASE* (*FADox*) (Brown and Jenkins, 2008).

A high dose of UV-B activates also stress integrator genes such as the mitogen-activated protein kinases (MPKs), MPK3 and MPK6, and their negative regulator MPK phosphatase1 (MKP1). The functionality of these stress integrators has been shown by genetic analyses in *Arabidopsis* with *mpk3* and *mpk6* mutants that were more tolerant while *mkp1* mutants were hypersensitive to UV-B radiation (González Besteiro et al., 2011; González Besteiro and Ulm, 2013). Higher doses of UV-B trigger largely the formation of cyclobutane pyrimidine dimers (CPDs) and to approximately 25% of damaged bases, pyrimidine [6-4] pyrimidone dimers ([6-4] photoproducts; [6-4] PPs) (Britt et al., 1993; Britt, 2004). However, photolyases rapidly repair these pyrimidine dimers during photoreactivation which needs minimal amounts of visible or at least UV-A (315–400 nm) or blue light. Higher doses of UV-B (4 µmol m^-2^ s^-1^) also induce the expression of the recombinase *RAS ASSOCIATED WITH DIABETES PROTEIN51* (*RAD51*) (Ulm et al., 2004; Lang-Mladek et al., 2012). RAD51 is recruited to sites of double-strand DNA breaks (DSBs) but also to promoters of defense genes (Yan et al., 2013). RAD51 associates with proteins involved in the repair by homologous recombination (Chapman et al., 2012).

Studies on the transcriptomic and proteomic level revealed that excess UV-B mediates cross-links between RNA and proteins within the ribosomes and cellular recovery is accompanied with increased transcription and translation of genes involved in protein synthesis (Casati and Walbot, 2004a). These include ribosomal proteins, initiation and elongation factors, and ribosome recycling factors. Furthermore, rapid and transient phosphorylation of the 40S ribosomal protein S6 (RPS6) and its S6 kinase was detected within 15 min of UV-B exposure in maize (Casati and Walbot, 2004a). RPS6 is involved in the selective translation of specific messenger RNAs (mRNAs) (preferentially ribosomal proteins and elongation factors). These mRNAs contain an oligopyrimidine stretch at the transcriptional start site (Meyuhas and Dreazen, 2009). Another group of proteins related to translation are members of the 80S ribosome, the RPL10 gene family. Quantification of protein synthesis upon UV-B exposure revealed that a heterozygous mutant of *Arabidopsis rpl10A* was hypersensitive to UV-B. While the rate of translation of wildtype and *rpl10B* and *rpl10C* mutants was reduced to 60% of control condition, it was even more affected in the heterozygous *rpl10A* after a 4 h exposure to UV-B (Ferreyra et al., 2010).

Apart of regulating translation at the ribosomal level, protein biosynthesis is controlled through a kinase phosphorylating the α-subunit of the Eukaryotic Initiation Factor 2 (eIF2). EIF2α is required for the delivery of the initiator tRNA^Met^ to the translation machinery. The evolutionary conserved protein kinase is *GCN2* (*general control nonrepressed 2/EIF2AK4*). GCN2 plays a central role in modulating protein biosynthesis in response to different environmental stresses causing a nutritional imbalance. GCN2 strongly reduces global protein synthesis *via* phosphorylation of eIF2α from yeast to mammals. In plants, GCN2 is activated in response to amino acid starvation, stimulated by herbicides such as glyphosate and chlorsulfuron, by purine deprivation through guanine alkylation with methyl methanesulfonate, by exposure to UV-C and low temperature, by wounding and the stress hormones methyl jasmonate and salicylic acid along with the ethylene precursor 1-aminocyclopropane-1-carboxylic acid (Lageix et al., 2008; Zhang et al., 2008; Faus et al., 2018). Recently, GCN2 has been assigned as carbon/nitrogen amino acid backbone sensor important for the biosynthesis of cysteine (Dong et al., 2017). Genetic analyses showed that *GCN2* is the only kinase phosphorylating eIF2α under diverse stress conditions in the model plant *Arabidopsis thaliana* (Lageix et al., 2008; Zhang et al., 2008; Faus et al., 2018).

The aim of this study was to evaluate whether and how UV-B is activating GCN2 and which signaling pathway might be involved. Since GCN2 is a central regulator of translation the rate of translation in *gcn2* mutants in ambient and UV-B enriched light was quantified as well as CPD formation and repair. Growth characteristics revealed an increased tolerance of *gcn2* mutants towards chronic exposure to UV-B which correlated with a reduced CPD formation. The role of *GCN2* in UV-B triggered inhibition of translation is supported by *gcn2* mutants exhibiting a higher rate of translation upon UV-B compared to the wildtype backgrounds. The higher tolerance of *gcn2* mutants towards UV-B might in part be due to the constitutive higher expression of *CHS*, an early gene in the phenylpropanoid pathway and the increased ability to protect *gcn2* mutants from DNA damage.

## Materials and Methods

### Plant Material

*Arabidopsis thaliana* Columbia accession (Col-0) and Landsberg *erecta* (Ler) were used as wild type controls. The T-DNA knock-out alleles in At5g18610, *gcn2-1* (GT8359) (Zhang et al., 2008) and *gcn2-2* which was purified from a second T-DNA insertion of SALKseq_032196 line (Faus et al., 2018) are in Ler and Col-0 background, respectively. The UV-B and stress signaling mutants *uvr8-6* (SALK_033468) (Favory et al., 2009), *cop1-4* (McNellis et al., 1994), *mpk6-2* (SALK_073907) (Nakagami et al., 2006) and *mpk3* (SALK_151594) (Nakagami et al., 2006) are in Col-0 background while *hy5-ks50* (Oyama et al., 1997), *hy5-ks50/hyh* (Holm et al., 2002), and *mkp1* (Ulm et al., 2001) are in Wassileskija (Ws).

### Growth Conditions, UV-C, and UV-B Treatments

Seeds were sterilized in 5% sodium hypochlorite as described previously (Benfey et al., 1993). Sterile seeds were plated on MS (Duchefa Biochemie) medium supplemented with 4.5% sucrose and 1% plant agar (Duchefa Biochemie). Seeds were stratified in the dark at 4°C for 48 h and transferred to a continuous light cabinet (RUMED, Rubath Apparate GmbH) with 22°C for 11–15 days. For experiments with soil grown plants, seedlings were transferred to soil (50% potting soil, 50% perlite) and cultivated at 20°C and 70% relative humidity (York, Austria) in a 16/8 h light/dark cycle (Philips TLD36W/840) of 75 µmol m^-2^ s^-1^ photosynthetically active radiation (PAR, 400–700 nm) until UV-B treatments or seed maturation. For UV-C treatments, 10 days old seedlings cultivated on solid MS plates were exposed for 20 min in a crosslinker (Hoefer, 254 nm, max. µJoules), recovered in liquid 1% MS medium supplemented with 1% sucrose for 1 h, snap frozen in liquid nitrogen and stored at –80°C. UV-B radiation started always 3 h after the onset of the day/night cycle (16 h light/8 h dark). For broad band UV-B exposure, approximately 25–30 days old soil grown plants were exposed for different times with 6–10 µmol m^-2^ s^-1^ (1.3–2.2 W/m^2^) under Philips TL20W/12RS tubes in a growth chamber with 140–150 µmol m^−2^ s^−1^ of white light provided by Philips F17T8/TL741 fluorescent tubes (Philips, Amsterdam, Netherlands) and two additional HB GroLED lamps (CLF Plant Climatics, Wertingen, Germany). For chronic UV-B treatments, soil grown plants of about 25 days were exposed to 140–150 µmol m^−2^ s^−1^ of white light supplemented for 1 h/day with 6 µmol m^-2^ s^-1^ UV-B for 15 days. A cellulose di-acetate filter (ULTRAPHAN Acetatfolien^®^, 0.05 mm, Wettlinger Kunststoffe) was placed between the plants and the broad band UV-B lamps for filtered UV-B treatments. Philips TL20W/01-RS tubes were used with 3.5 µmol m^-2^ s^-1^ for narrow band UV-B treatments (spectra in **Supplementary Figure S1**). Fluence rates of white light (PAR) were measured using a Black-Comet C-UV/VIS spectrometer (StellarNet, Inc., Carlson, FL) with a SKU435 UV-B sensor and the SPECTRAWIZ^®^ Software (Mainz, Germany). The UV-B dosage was regulated by an Apogee UV-Sensor UVS (Model SU-100) positioned at the height of the rosette leaves about 40 cm below the UV-B tubes.

### Evaluations of Phenotypes

For the phenotypical evaluations rosette diameter, stem length and seed weight were quantified during and at the end of the chronic UV-B treatments. The rosette diameter was measured after 10 and 15 days at three positions of each rosette. Stem lengths were measured after stopping watering, when the plants were almost dry (around 12–15 days after the end of the UV-B treatment). The seeds were harvested from completely dry plants and weighed.

### Western Blots for GCN2 Activity With Phospho-eIF2α (Ser51) Specific Antibodies

Approximately 300 mg of leave tissue was ground with liquid nitrogen and resuspended with ice-cold 500 µl eIF2α extraction buffer [25 mM Tris/hydrochloride (Tris/HCl) pH 7.5, 75 mM sodium chloride, 5% glycerol, 0.05% Triton-X-100, 0.5 mM ethylenediaminetetraacetic acid (EDTA) pH 8.0, 0.5 mM egtazic acid pH 8.0, 2 mM dithiothreitol, 2% polyvinyl pyrrolidone containing protease (complete mini EDTA-free; Roche) and phosphatase inhibitors (20 mM β-glycerolphosphat, 0.1 mM sodium orthovanadate, 25 mM sodium fluoride]. After centrifugation (Eppendorf centrifuge 5430R) for 30 min at 4°C and 15,000 g the supernatant was transferred to a new tube and centrifuged again for 15 min with the same settings. This supernatant was stored at -80°C. After quantification with the Qubit Protein Assay Kit and the Qubit Fluorometer (both Invitrogen/Molecular Probes) 5× sodium dodecyl sulfate polyacrylamide gel electrophoresis (SDS-PAGE) loading buffer was added to 20 µg protein and separated without prior heating on a 10% SDS-PAGE with 20 mA until the blue marker reached the end of the gel. After blotting the immunodetection was performed using 1:2,000 diluted Phospho-eIF2α (Ser51) antibody (Cell Signaling Technology; #9721) and 1:10,000 diluted secondary ECL anti-rabbit IgG horseradish peroxidase antibody (GE Healthcare).

### Quantification the Rate of Global Protein Synthesis With Puromycination Assays

The rate of global protein synthesis was quantified with puromycin (PU) labeled nascent proteins and detection of the incorporated PU by Western blots. Fifteen 10 days old seedlings were transferred into six-well plates with sterile water and puromycin dihydrochlorid (Carl Roth) was added to a final concentration of 65 µg ml^-1^ if not otherwise specified. After PU incubation for 2 h in the continuous light cabinet (RUMED, Rubath Apparate GmbH) seedlings were weighed and flash frozen in liquid nitrogen. Extraction buffer (25 mM Tris/HCl, pH7.5, 50 mM potassium chloride, 5 mM magnesium chloride, 5 mM dithiothreitol, 0.5 mM phenylmethylsulfonyl fluoride) was added to pulverized plant material in a ratio 1:1 (microliter/milligram), vortexed thoroughly and solid residues were separated by centrifugation for 15 min at 13,000 g and 4°C. Protein concentration of the supernatant was quantified with the Qubit system (Invitrogen). Fifteen micrograms of total protein were separated on 10% SDS-PAGEs. For Western blot, proteins were transferred onto polyvinylidene difluoride membranes (Carl Roth). A second gel served as loading control and was stained over night with Coomassie Brilliant Blue [0.25% (w/v) in 45% ethanol/10% acetic acid]. For dot blots, 1 µl of serial protein dilutions (1, 0.8, 0.64, 0.32, 0.16 µg µl^-1^) were dropped on a dry nitrocellulose membrane (Roth, Germany) in triplicates. Membranes were first dried at room temperature for at least 20 min and subsequently baked at 80°C for 2 h pressed between two glass plates with filter papers in between. Western and dot blot membranes were blocked with 5% milk powder in Tris-Buffered Saline, 0.1% Tween (TBS-T). Incorporated PU was immunodetected with 1:10,000 dilutions of mouse anti- antibodies for at least 3 h (MABE343 clone 12D10, Merck Millipore, Darmstadt, Germany) and 1:10,000 diluted goat anti-mouse HRP-conjugated (New England Biolabs GmbH, NEB, Frankfurt am Main, Germany) secondary antibodies in TBS-T. Signal detection was done with the Roti-Lumin-Plus substrate (Carl Roth, Germany) and digitalized in the Fusion Pulse TS (Vilber, Germany). For normalization, Western blot membranes were washed after immunodetection again with TBS-T and total proteins were visualized with Ponceau S stain [0.5% (w/v) Ponceau S in 1% acetate; Carl Roth, Germany] and Coomassie Brilliant Blue [0.1% (w/v) in 40% ethanol/10% acetic acid]. After de-staining with water or 40% ethanol/10% acetic acid, respectively, membranes were dried and the signal detection was performed with the ChemiDoc XRS+ (Bio-Rad). The rate of translation was determined by measuring the signal intensities of all lanes of Western blots and Coomassie stained gels as well as dots from dot blots with the EvolutionCapt software (Vilber, Germany) using rolling ball background subtraction. Signals of total proteins stained with Ponceau S or Coomassie Brilliant Blue on membranes were quantified with the Image Lab Software 5.1 (Bio-Rad) using local background subtraction. The volumes of the PU signal of each lane or dot were divided by the adjusted volumes of total protein signal and the input protein, for dot blots respectively. To compare experimental repetitions the data were normalized to the mean of the control conditions of each blot.

### RNA Isolation, cDNA Synthesis, and Quantitative Real-Time PCR

Total RNA isolation, complementary DNA (cDNA) synthesis and quantitative real-time polymerase chain reaction (RT-qPCR) were performed as previously described (Karsai et al., 2002; Lang-Mladek et al., 2012). Primer pairs used for amplification are listed in **Supplementary Table S1**. RT-qPCR was performed on a Rotor-Gene 3000 (Corbett, Qiagen, Hilden, Germany) in 14 µl reactions containing 5 pmol of each gene specific primer, 1 µl 1:10 diluted cDNA and the 5× HOT FIREPol EvaGreen^®^ qPCR Mix Plus (Solis Biodyne, Tartu, Estonia). In total four different experiments (i.e. biological repeats) were quantified. Each cDNA was measured in triplicate. Amplification occurred after an initial denaturation (15 min/94°C) in 40 cycles (94°C/5 s – 54°C/5 s – 66°C/25 s + acquisition – 81°C/15 s acquisition – 85°C/15 s acquisition). To determine the PCR efficiencies of each run, a dilution series for each analyzed gene(s) was included. Melting curves were recorded between 65 and 99°C at the end of each run. Gene expression was calculated using the efficiencies of each gene with the RotorGene software (Version 6.0) and Excel (Pfaffl, 2001; Bustin et al., 2009). Relative expressions were normalized to the three reference genes (**Supplementary Figure 2**), the regulatory subunit of *PROTEIN PHOSPHATASE2* (*PP2A*), *TUBULIN BETA9* (*TUB9*), and *UBIQUITIN5* (*UBQ*) (for primer, fragment size and efficiencies see **Supplementary Table S1**).

### DNA Damage Analyses

Plants were treated for 1 h with broad band UV-B and whole rosettes were harvested in 15 ml tubes immediately or after 4 h recovery and flash frozen. Genomic DNA was isolated with hexadecyltrimethylammonium bromide (CTAB) as follows: frozen material was ground in liquid N_2_, suspended in 5 ml 2× CTAB buffer [2% (w/v) CTAB, 100 mM Tris/HCl pH 8.0, 20 mM EDTA, 1.4 M sodium chloride, 1% (w/v) polyvinylpyrrolidone PVP40] and incubated for 20 min at 65°C. After cooling on ice, 5 ml chloroform was added and vortexed for 2 min. After centrifugation with 7,500 g at 5°C for 5 min the upper phase was transferred into a new tube and the DNA was precipitated with 4 ml isopropanol at room temperature. DNA was pelleted for 20 min at 5°C and 7,500 g, washed with 500 µl 70% ethanol and dissolved in 100 µl 1× Tris-EDTA (pH 7.5) and 3 µl DNase free RNase A (Carl Roth, Germany). DNA was quantified using the Qubit system (Invitrogen) and solutions containing 396 ng DNA in 82.5 µl of 1× Tris-EDTA were prepared. DNA was denatured by adding 0.6 N sodium hydroxide to a final concentration of 0.3 N and incubated at room temperature for at least 10 min. A Roti^®^-Nylon plus (Carl Roth, Germany) membrane was soaked in water for 15 min. After assembling the Bio dot microfiltration apparatus (Bio-Rad) the membrane was washed with 200 µl distilled water per well. The samples were applied in 25 µl aliquots with six technical repeats (60 ng), then washed twice with 200 µl of 0.4 N sodium hydroxide. The membrane was dried completely at room temperature and subsequently baked at 80°C for 2 h in-between filter papers and glass plates. For immundetection of the UV-B induced CPDs, the membrane was blocked for 1 h with 5% milk powder in TBS-T. CPDs were detected with 1:2,000 mouse anti-CPD antibodies (Cosmo Bio Co., Ltd, Japan) over night at 5°C and 1:4,000 goat anti-mouse-HRP (NEB) for 1 h as secondary antibody. Signal detection was done with the Roti^®^-Lumin plus substrate (Carl Roth, Germany) and digitalized with the ChemiDoc XRS+ (Bio-Rad). The signal intensities were quantified with the Image Lab Software 5.1 (Bio-Rad) using global background subtraction.

### Statistical Analysis

The data was analyzed with Excel and is presented as means +/- standard error (SE). Statistical significant differences between the wildtype, *gcn* mutants in respect to phenotypes, DNA damage and rate of protein synthesis were determined by Student’s t- test and p < 0.05 were marked with stars. One-way analysis of variance (ANOVA) was used for the DNA damage analyses.

## Results

### Broad Band UV-B Radiation Activates GCN2

**Figure 1:**
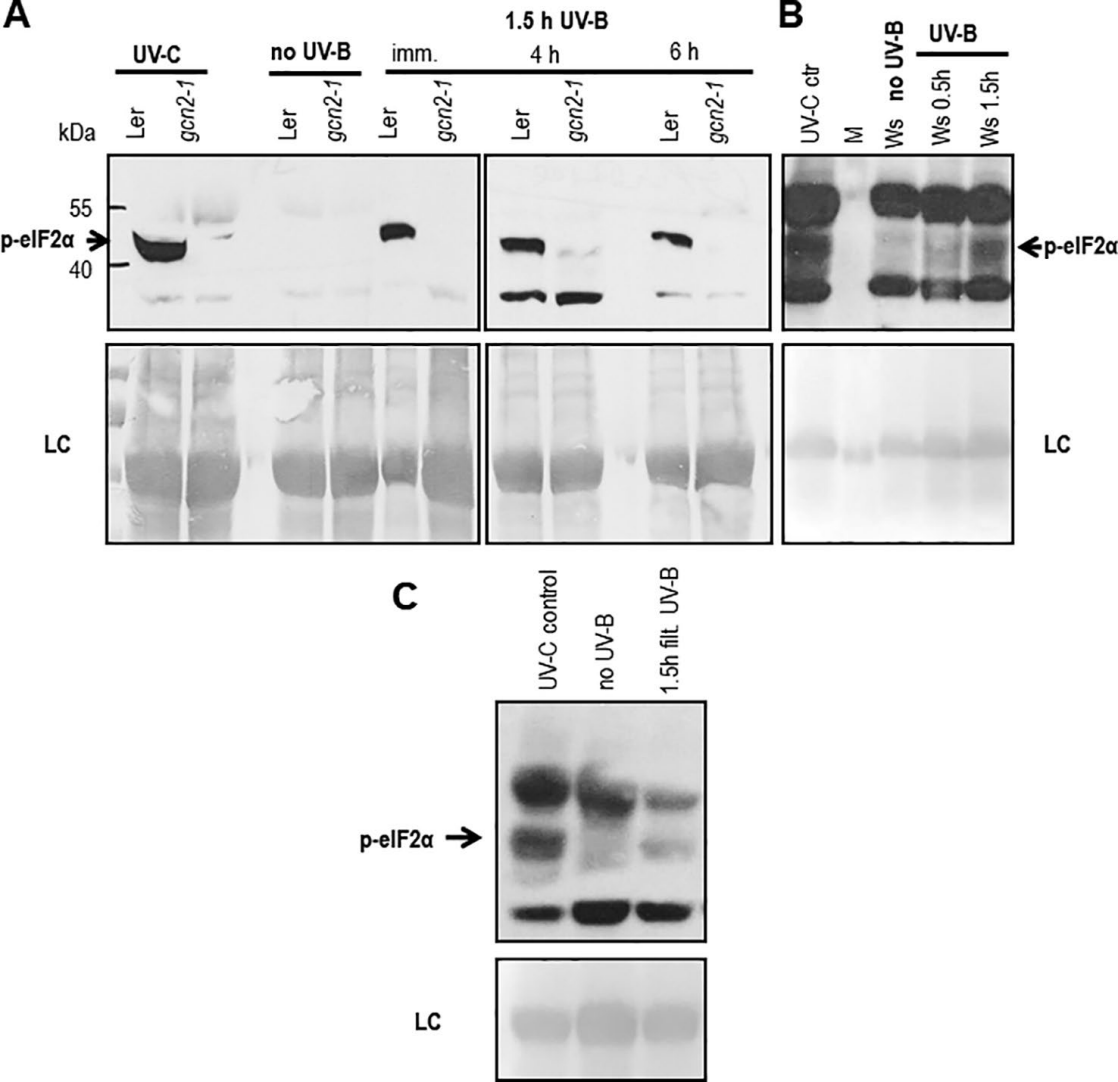
Western blots assessing the activation of GCN2 *via* eIF2α phosphorylation. **(A)** Wildtype (Ler) and *gcn2-1* plants were either treated 20 min with UV-C (control) or with white light supplemented for 1.5 h with 8 µmol m^-2^ s^-1^ broad band UV-B. Leaves were harvested immediately (imm.), 4 h and 6 h after UV-B shut down. **(B)** First signs of eIF2α phosphorylation (p-eIF2α) is detectable already 30 min after the onset of 10 µmol m^-2^ s^-1^ broad UV-B. **(C)** Also 1.5 h of filtered broad band UV-B exposure is activating GCN2. Equal amount of protein (20 μg) was loaded on 10% sodium dodecyl sulfate polyacrylamide gel electrophoresis. LC, Loading control.

In *Arabidopsis*, several studies confirmed GCN2 activation after exposure to UV-C, however little is known about the biologically more relevant UV-B radiation. To investigate the role of GCN2 in responses to UV-B its activity was analyzed through eIF2α phosphorylation assays in wildtype and *gcn2-1* mutants. First we determined whether eIF2α was detectably phosphorylated upon broad band UV-B in wildtype. Indeed eIF2α phosphorylation is evident after a 90 min exposure to 8 µmol m^-2^ s^-1^ and even 6 h after UV-B shut down. Since in *gcn2-1* mutants eIF2α phosphorylation under these conditions is absent GCN2 is the only kinase responsible (**Figure 1A**). We next determined whether cellulose diacetate filtered UV-B stimulates eIF2α phosphorylation and when eIF2α phosphorylation is detectable after the start of the UV-B exposure (**Figures 1B, C**). First signs of eIF2α phosphorylation were detectable already 30 min after the onset of broad band (**Figure 1B**) and filtered broad band UV-B (**Figure 1C**). However, eIF2α phosphorylation was neither detectable immediately after a 1.5 h UV-B exposure nor 2 h after shut down of the narrow band lamps with a wave length maximum of 311 nm (**Supplementary Figure S5**). These findings indicate that the activation of GCN2 lies between 290 and 308 nm.

### GCN2 Activation Is Independent of the UV-B Photoreceptor and the Stress Signaling Kinases MPK3 and MPK6

**Figure 2:**
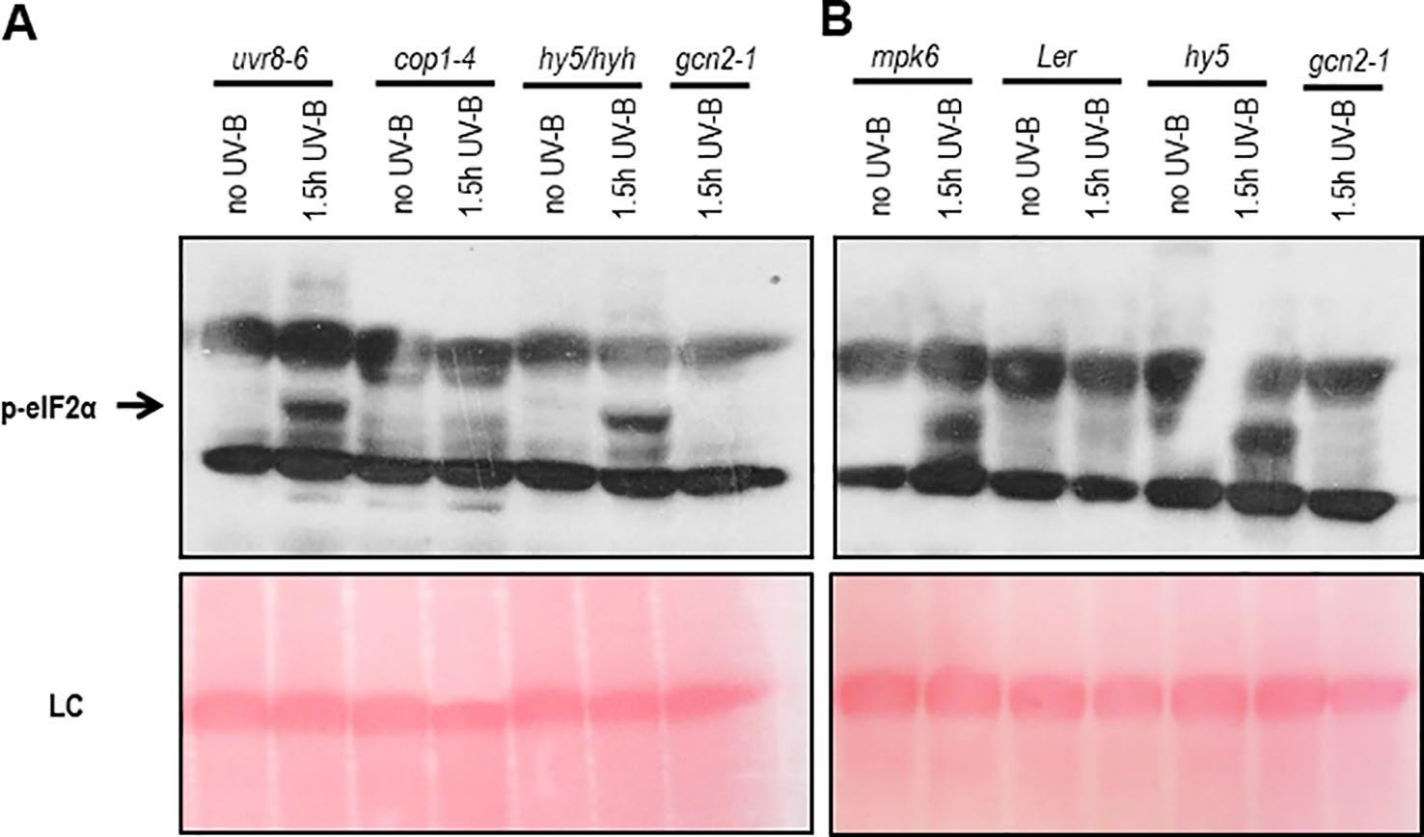
Western blots assaying activation of GCN2 *via* eIF2α phosphorylation in UV-B specific and stress signaling mutants. **(A)** Mutants of the UV-B photoreceptor specific signaling pathway and *gcn2-1* were harvested after 1.5 h of 6 µmol m^-2^ s^-1^ broad band UV-B treatment. **(B)** Wildtype and mutants of the stress and UV-B signaling pathway after 1.5 h of broad UV-B with 10 µmol m^-2^ s^-1^. Equal amount of protein (20 μg) was loaded on 10% sodium dodecyl sulfate polyacrylamide gel electrophoresis. LC, Loading control.

To determine whether the activation of GCN2 depends on the UVR8-COP1-HY5/HYH or a general, but different UV-B induced stress signaling pathway, a genetic approach was exploited. For the UV-B specific signaling pathway eIF2α phosphorylation was assessed in *uvr8-6* mutants and mutants of the key light regulator and UVR8 interaction partner *cop1-4* and the downstream transcription factors *hy5* and *hyh* (**Figures 2A, B**). In all these UV-B photoreceptor dependent mutants eIF2α phosphorylation was detected. Since the dose of UV-B sufficed to activate the UV-B stress response pathway eIF2α phosphorylation was also examined in mutants of the MAP kinases, *mpk3* and *mpk6* and their negative regulator *mkp1*. Independent if the broad band UV-B was filtered (data not shown) or not (**Figure 2B** and **Supplementary Figure S4B**) eIF2α phosphorylation was detectable in these stress signaling pathway mutants demonstrating that the activation of GCN2 is neither triggered by the UVR8-COP1-HY5/HYH nor the MAP kinases stress signaling pathway.

### UV-B Related Genes Are Differentially Expressed in *gcn2* Mutants

**Figure 3:**
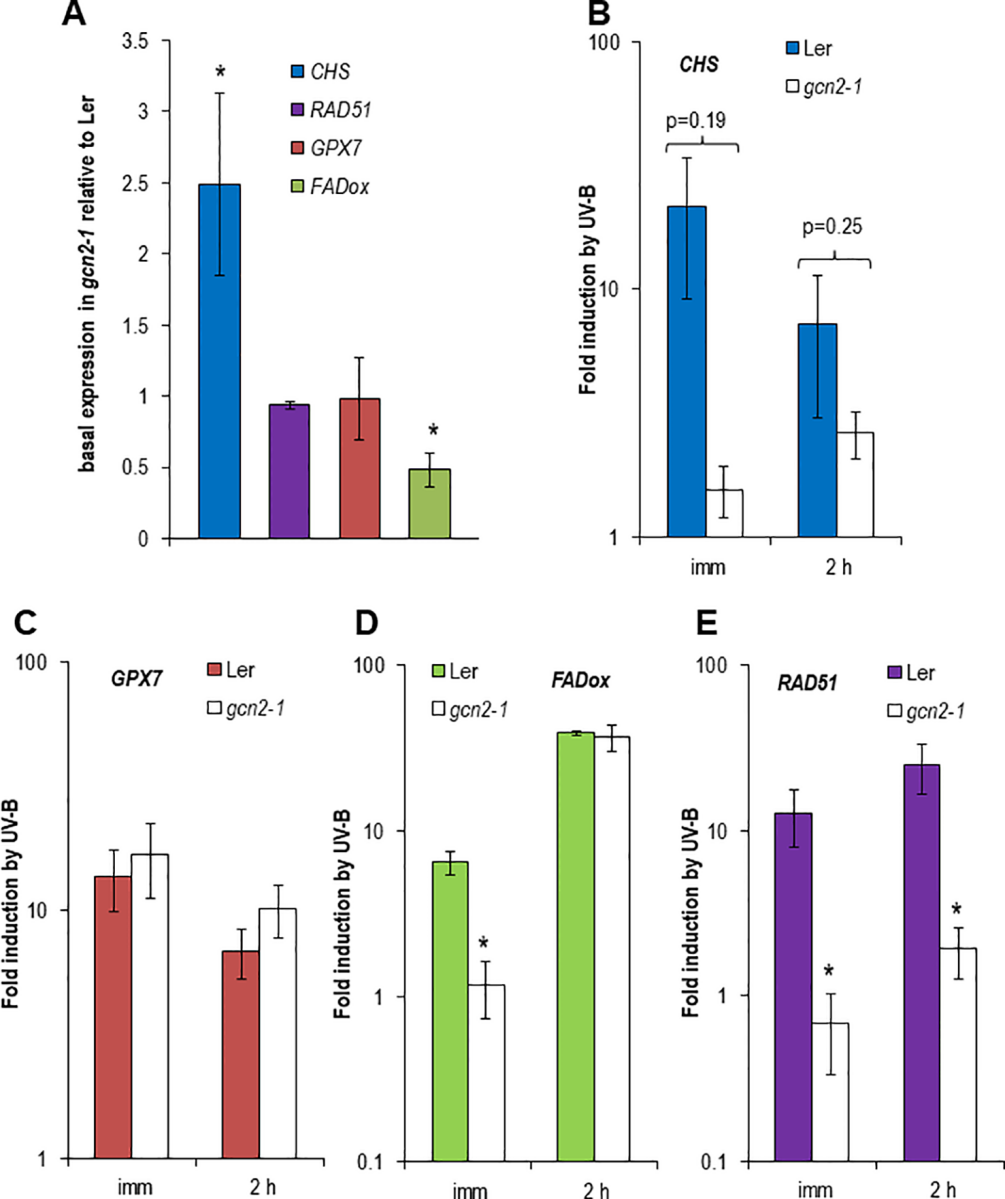
Expression of reporter genes dependent or independent of UVR8 and indicative for DNA damage in wildtype Ler and *gcn2-1*. In total, the expression of four different experiments (i.e. biological repeats) was quantified. Each cDNA was measured in triplicate. Data represent the mean and standard errors of the normalized expression to three reference genes. **(A)** Basal expression relative to the wildtype Ler using normalized data of three reference genes. **(B**–**E)** Fold induction by UV-B immediate and 2 h after a 1.5 h exposure to broad band UV-B of **(B)** the UVR8 dependent *CHS*, **(C)** the UVR8 dependent *GPX7*, **(D)** the UVR8 independent *FADox* and **(E)** the DNA damage induced *RAD51*. Stars indicated significant different expression to wildtype.

UV-B is inducing the expression of specific genes which are indicative for different signaling pathways. Among them is the gene for the first enzyme in the flavonoid biosynthesis, the CHS synthase. CHS is induced in an UVR8-COP1-HY5/HYH dependent manner at low UV-B fluence rates but also at higher and stressful UV-B fluence rates (Brown and Jenkins, 2008; Lang-Mladek et al., 2012). *GPX7* also depends on the UVR8-COP1-HY5/HYH pathway and similarly to all glutathione peroxidases it is involved in the protection against photooxidative stress (Chang et al., 2009). In contrast, *FADox* is UVR8-COP1-HY5/HYH independent and plays a role in the biosynthesis of 4-hydroxyindole-3-carbonyl nitrile, a metabolite with cyanogenic function and important for pathogen defense (Brown and Jenkins, 2008; Rajniak et al., 2015). Since in our experiments unfiltered broad band UV-B with comparable to outdoor fluence rates was used the DNA damage responsive gene, *RAD51*, was included in the quantitative expression analyses. Surprisingly the *CHS* gene was constitutively higher expressed in *gcn2-1* mutants compared to wildtype while the *FADox* expression was significantly lower and *GPX7* and *RAD51* had similar expression levels to wildtype under control conditions (**Figure 3A**). *CHS* induction upon to UV-B exposure was weaker and delayed in *gcn2-1* compared to wildtype (**Figure 3B**), while *GPX7* behaved similar to wildtype (**Figures 3C, D**). Similar to the *CHS* gene also the induction of *FADox* expression was delayed in *gcn2-1* mutants but reached 2 h after UV-B shut down a comparable level as wildtype. A rather unexpected surprise was the expression behavior of the DNA damage reporter gene *RAD51*. While in wildtype *RAD51* was about 12 to 25 fold induced upon UV-B exposure this was not the case in *gcn2-1* mutants (**Figure 3E**). *RAD51* induction upon UV-B exposure was delayed in *gcn2-1* compared to wildtype and never reached the expression level of wildtype (**Figure 3E**). These expression analyses indicate that the DNA damage signal is weaker in *gcn2-1* mutants. One might speculate that the constitutive higher expression of a key gene in the phenylpropanoid biosynthesis pathway, *CHS*, is involved in the accumulation of UV-B scavenging components, protecting *gcn2-1* mutants from excess UV-B and therefore delaying and weakening the typical transcriptional responses to UV-B.

### *Gcn2* Mutants Develop Less Cyclobutane Pyrimidine Dimers Upon UV-B but the Repair Is Similar to Wildtype

**Figure 4:**
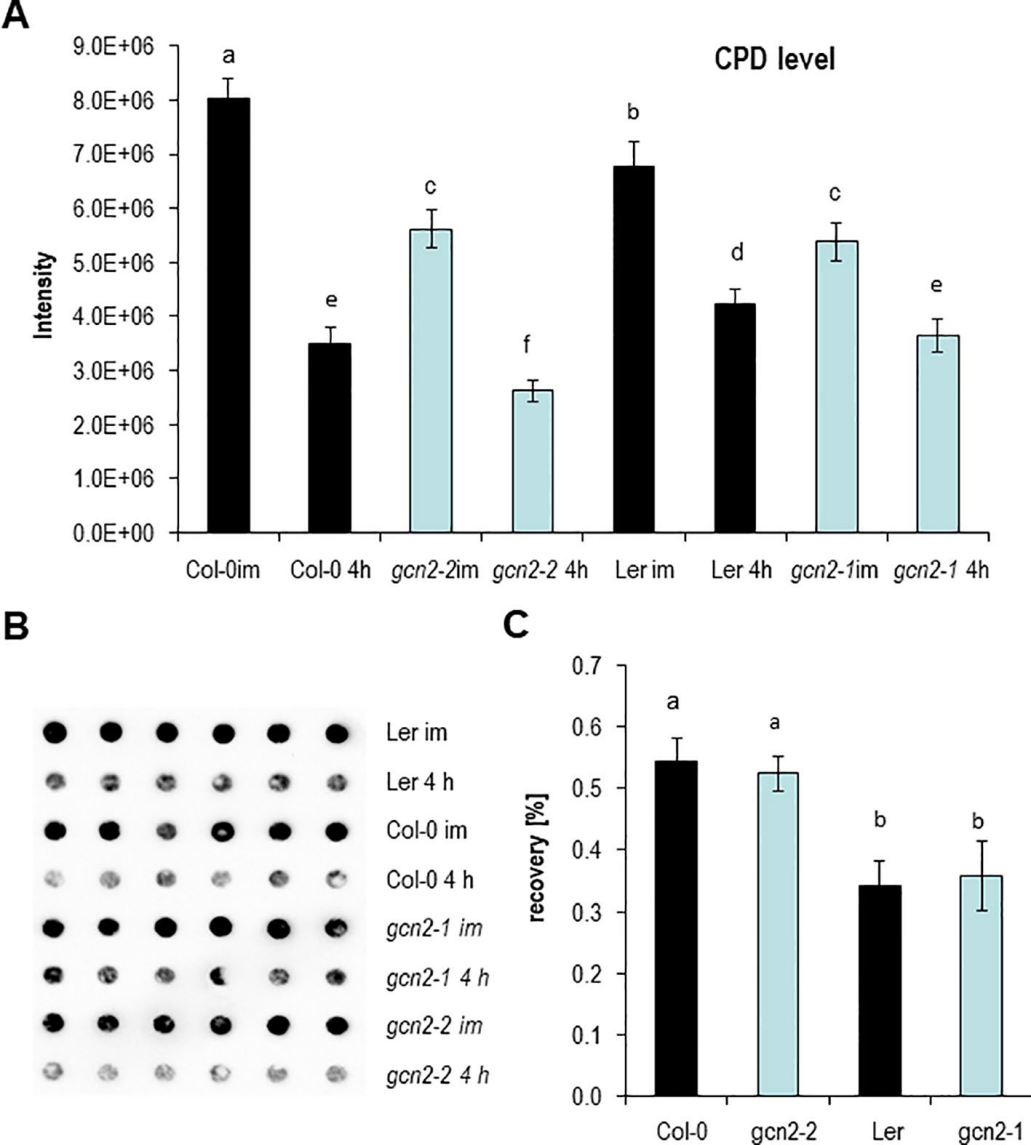
DNA damage and repair analysis. **(A)** Absolute CPD levels of plants harvested immediately after 1 h UV-B exposure and 4 h after UV-B shut down. **(B)** Representative image of a genomic DNA dot blot hybridized with anti-CPD antibodies. **(C)** Calculated recovery after UV-B exposure. Graphs represents means and ± SE of three to five experiments (six dots per experiment). Letters indicate the significant differences.

Encouraged by the *RAD51* expression data, the level of UV-B induced CPDs were quantified in *gcn2* mutants and compared to wildtype with dot blot assays. For these analyses rosettes of soil grown plants of two *gcn2* alleles with different wildtype backgrounds (*gcn2-1* in Ler, *gcn2-2* in Col-0) were treated for 1 h with UV-B supplemented to white light and harvested immediately. To assess photorepair, a similar amount of rosette leaves were harvested 4 h after UV-B shut down. Overall Ler accumulated significantly less CPDs than Col-0 (**Figure 4A**). Furthermore, wildtype plants accumulated more CPDs compared to *gcn2* mutants (**Figure 4A**). The difference between CPDs immediately and 4 h after shut down of UV-B was used to calculate the recovery due photorepair (**Figures 4B, C**). Photorepair (recovery) was more effective in Col-0 than Ler (**Figures 4A, C**). The mutants exhibited no differences in their rate of photorepair compared to the respective wildtype backgrounds (**Figure 4C**). These results indicate that stress activated GCN2 inhibits properties that support DNA protection upon UV-B exposure while CPD repair through photolyases is not differentially regulated between wildtype and *gcn2* mutants.

### The Rate of Translation Is Higher in *gcn2* Mutants

**Figure 5:**
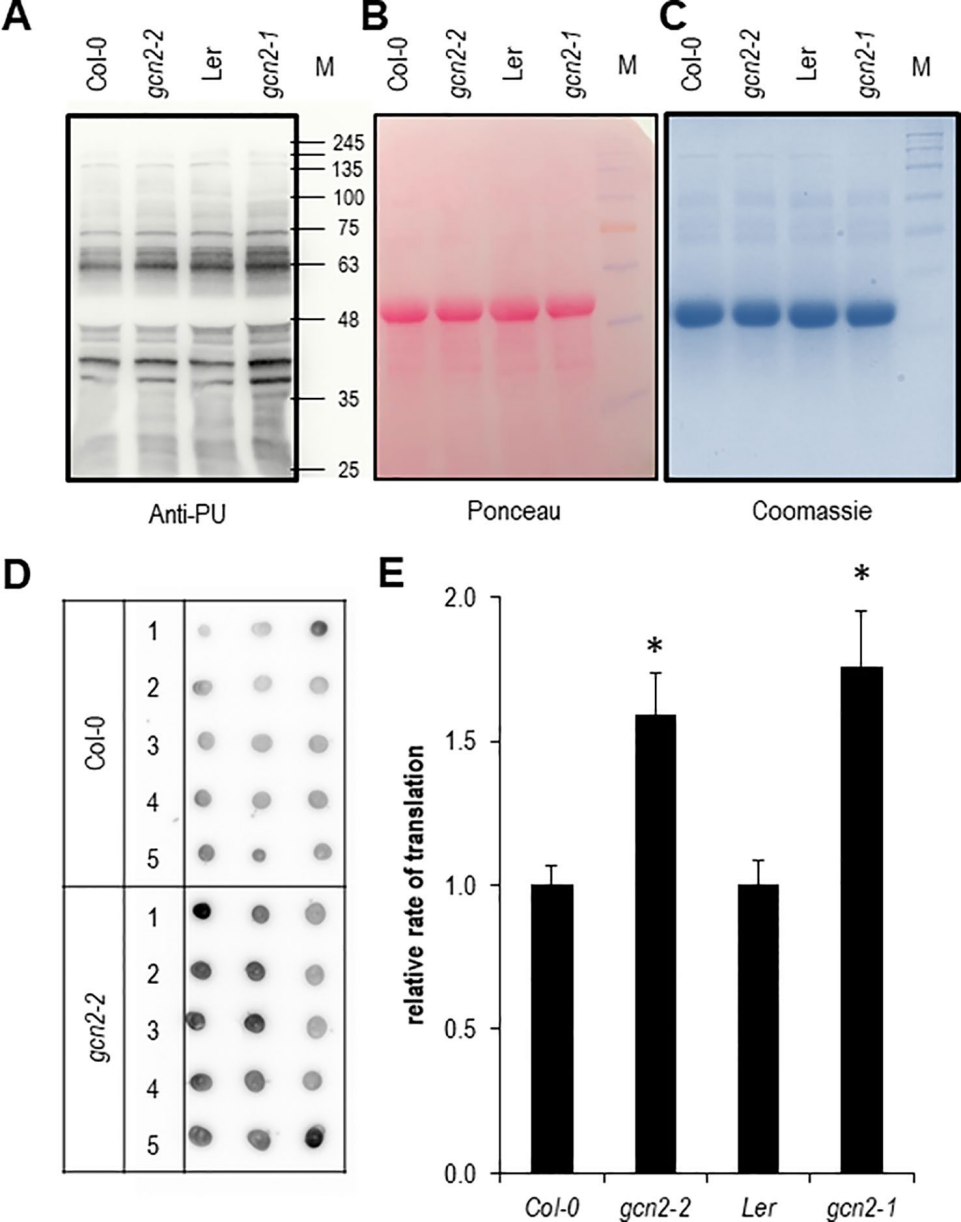
Quantification of the rate of protein biosynthesis using puromycin (PU) **(A)** Anti-PU Western blot with wildtype and *gcn2* mutants. To confirm equal loading of the 10% sodium dodecyl sulfate polyacrylamide gel electrophoresis, the membrane was stained with **(B)** Ponceau S and **(C)** Coomassie. **(D)** Example of a dot blot analysis with total protein extracts of PU treated seedlings where PU incorporation was detected with anti-PU antibodies. **(E)** Quantification of the dot blot analyses. Bars represent means and SE of at least 15 dots per experiment. Data of Col-0 and *gcn2-2* are means of three independent experiments, data from Ler and *gcn2-1* from a single experiment. Stars indicate significant differences (p < 0.05) between wildtype and mutants calculated with Student’s t- test. M designates the protein size marker line.

Next we aimed to quantify the rate of translation in *gcn2* mutants in comparison to their wildtype backgrounds by employing both Western and dot blot analyses with the non-radioactive puromycination assay (**Figures 5A–E**). The puromycination or SUface SEnsing of Translation method has been developed in mammalian cells and works also with plants (Schmidt et al., 2009; Van Hoewyk, 2016). A modified method called PU-associated nascent chain proteomics was used to directly monitor translation with a proteomic approach (Aviner et al., 2013; Aviner et al., 2014). In summary, multiple experiments have shown that the puromycination assay is a valid fast and cost-effective non-radioactive alternative to the classic ^35^S methionine/cysteine labeling methods for monitoring and quantifying the rate of global protein synthesis. As expected for the role as negative regulator of translation both *gcn2* mutants had a significantly higher rate of protein synthesis (**Figure 5E**).

### UV-B Reduces the Rate of Translation to a Lesser Extent in *gcn2* Mutants

**Figure 6:**
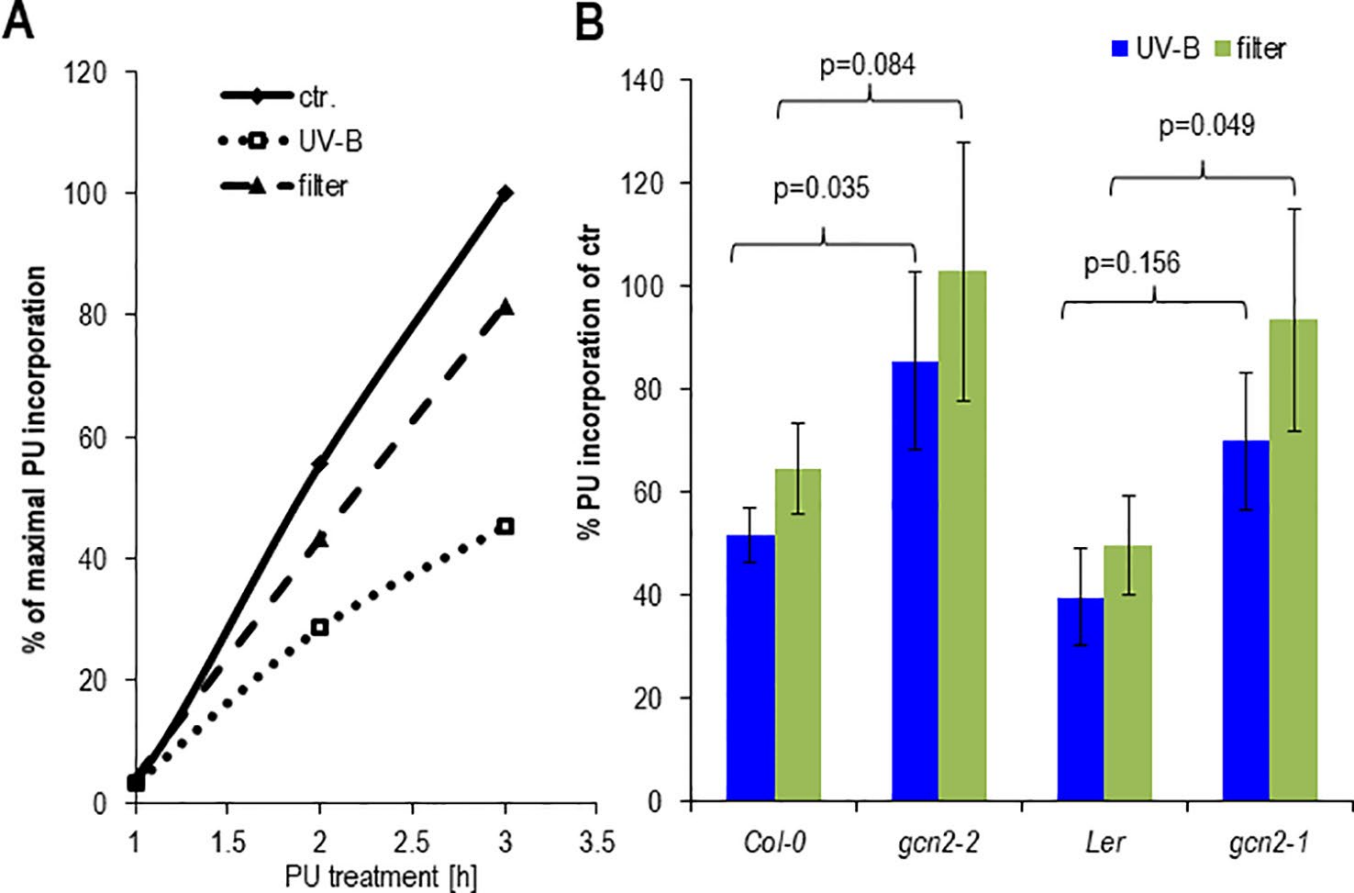
Effects of cellulose acetate filtered and unfiltered UV-B radiation on protein biosynthesis. **(A)** Time course of PU incorporation of detached rosette leaves. One hundred milligrams per milliliter PU was added at the onset of a 1 h UV-B treatment. Control leaves were only exposed to white light. Leaves were harvested either immediately after UV-B shut down which correspond to 1 or 2, and 3 h after PU addition. **(B)** Comparison of PU incorporation of wildtype and *gcn2* mutants upon UV-B exposure with filtered and unfiltered broad band lamps. In these experiments 100 µg/ml PU was added immediately after UV-B shut down and plant material harvested 1 h later. The graph represents the results of at least three independent Western blots. Error bars represent SE. P-values of student’s t-test are indicated above the brackets.

It has been shown that the rate of polysomal loading and thus translation adapts to various environmental changes among them light (Juntawong and Bailey-Serres, 2012; Liu et al., 2012; Pal et al., 2013). To determine the effect on protein biosynthesis of unfiltered and filtered UV-B, rosette leaves of soil grown plants or seedlings were exposed together with PU for 1 h to UV-B and puromycylation was quantified at different time points (**Figure 6A**). Both UV-B treatments resulted in less PU incorporation into newly synthesized proteins compared to only white light controls. Protein synthesis decreased by about 20% and 60% after a 1 h exposure to filtered and unfiltered UV-B and a PU labeling period of 3 h, respectively (**Figure 6A**). Although Col-0 wildtype and the *gcn2-2* mutant seemed to maintain a higher rate of PU incorporation and thus translation as Ler and *gcn2*-1, this accession specific effect was not significant (**Figure 6B**). The p-values were between Col-0 and Ler under unfiltered UV-B p = 0.2003, and under filtered UV-B p = 0.2688, and between *gcn2-2* and *gcn2-1* under unfiltered UV-B p = 0.6147 and under filtered UV-B p = 0.785. The results are consistent with experiments of *Arabidopsis* leaves exposed for 4 h to filtered UV-B of similar intensity and quantification of the rate of translation through *in vivo* [^35^S]Met labeling (Ferreyra et al., 2010). The reduced rate of translation recovered rapidly (**Supplementary Figure S3**) indicating the fast and dynamic response of translation to changing UV-B and light conditions. Similar to the no UV-B control condition both *gcn2* alleles maintained a higher rate of translation upon filtered and unfiltered UV-B exposure.

### *Gcn2* Mutants Are More Tolerant to UV-B

**Figure 7:**
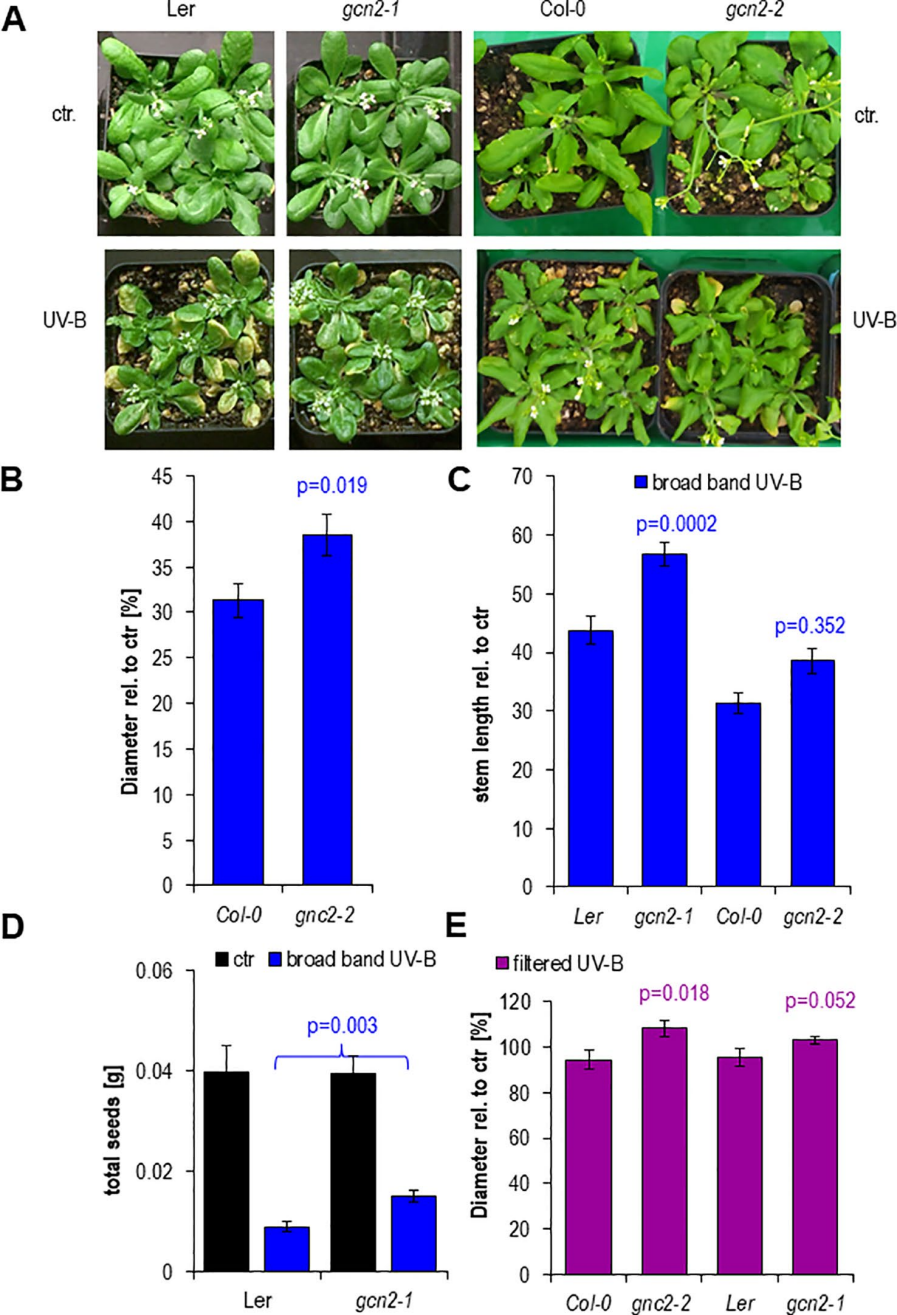
Phenotypes after 15 days of chronic UV-B exposure. **(A)** Rosettes of wildtype and *gcn2* mutants after a 1 h daily dose with 6 µmol m^-2^ s^-1^ UV-B broad band UV-B. **(B)** Quantification of rosette diameters of 18 to 21 plants. **(C)** Quantification of the stem lengths of 9 to 20 plants. **(D)** Quantification of the total seed weight of 9 to 19 plants. **(E)** Quantification of rosette diameter under chronic filtered broad band UV-B of 10 to 25 plants. Bars represent means and SE. Statistical differences between wildtype and mutants were calculated with Student’s t- test and p-values are indicated above each bar. Numbers of plants are designated in the respective bars.

Finally we examined the functional relevance of the transient misregulation of translation in *gcn2* mutants on growth parameters such as rosette size (diameter), stem length and fecundity quantified *via* the total seed weight (**Figures 7A–E**). Rosettes of both *gcn2* alleles developed larger under a daily exposure to filtered and unfiltered broad band UV-B compared to their respective wildtype accessions (**Figures 7C, E**). A similar difference was quantified with stem length, although only the Ler accession allele *gcn2-1* was significantly higher than wildtype (**Figure 7C**) probably because the Col-0 accession grew generally larger than Ler and did not reach their final height. Highly significant was the effect on total seed weight (**Figure 7D**). These results clearly demonstrate the importance of translational control. They also illustrate, that even transient misregulations, for example through a daily dose of 1 h elevated UV-B, generate dramatic effects on growth, the overall development and fecundity.

## Discussion

**Figure 8:**
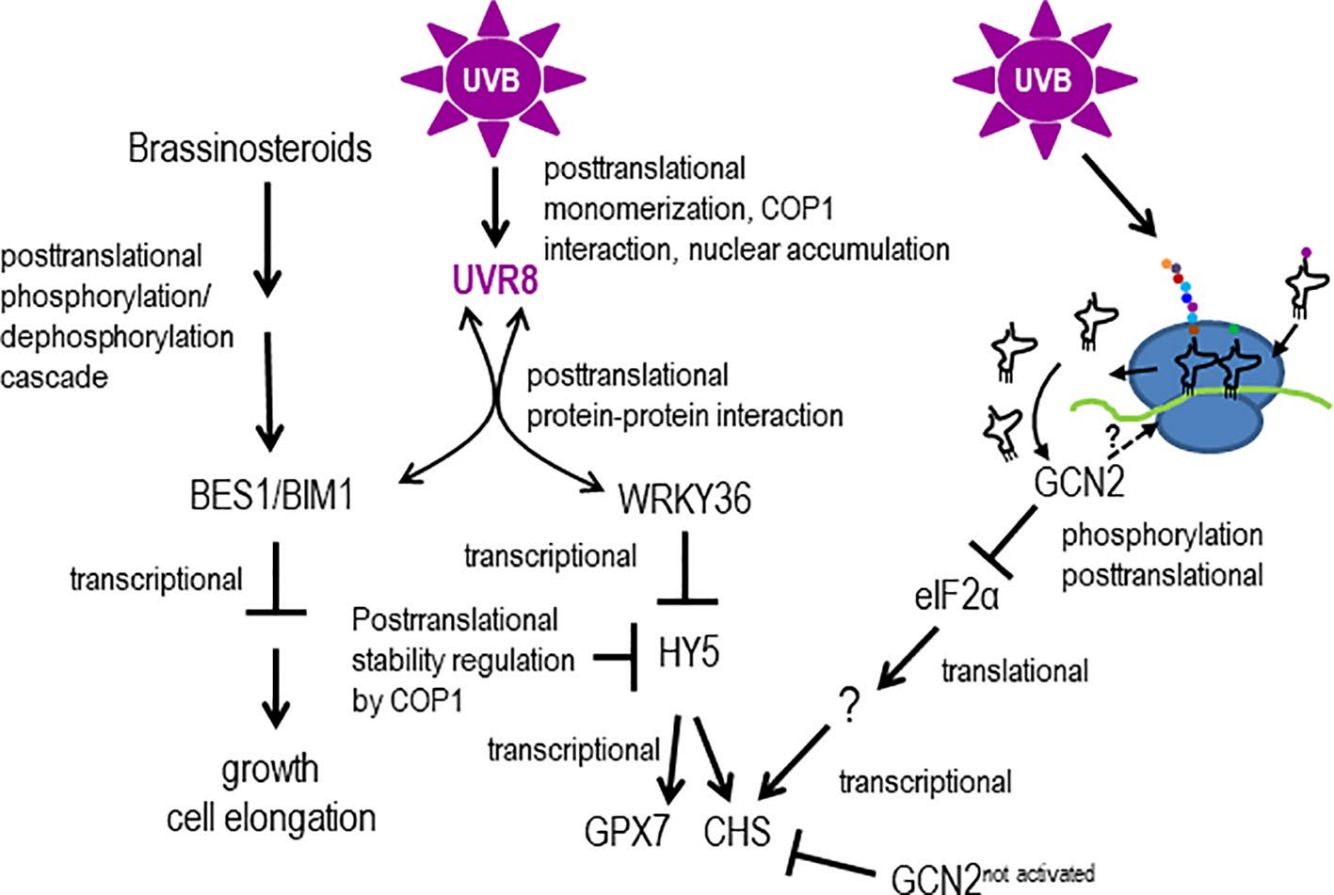
Models hypothesizing, how GCN2 might regulate UV-B responses. UV-B responses are mediated by transcriptional and (post)translational control involving the UV-B photoreceptor UVR8 or not. UV-B triggers UVR8 monomerization, interaction with COP1 and changes of the subcellular compartment. In the nucleus, UVR8 directly interacts with the downstream transcription factors BES1/BIM1 and WRKY36. Interaction with BES1/BIM1 and WRKY36 titrates these transcription factors away from promoters of genes involved in cell elongation and growth and allows HY5 to bind to its own and UV-B responsive promoters. Unbound COP1 destabilizes HY5. UV-B induces posttranslationally the activity of GCN2 which might be activated through structural changes of ribosomes by RNA-protein crosslinking, overaccumulation of uncharged tRNAs or direct ribosome binding. Activated GCN2 phosphorylates eIF2α and thereby changing the population of translated mRNAs. Not activated GCN2 might indirectly repress the transcription of energy demanding biosynthesis genes such as *CHS*.

Our GCN2 activation experiments show that eIF2α phosphorylation is detectable already after 30 min of UV-B between 290 nm and 308 nm and thus belongs to the early UV-B induced events being faster than most gene expression responses (Kilian et al., 2007). The GCN2 activation is independent of the UVR8-COP1-HY5/HYH and the MPK3, MPK6 and MKP1 stress signaling pathway. This poses the question which other signaling pathways might trigger GCN2. Recently it has been shown that UVR8 directly interacts apart from the E3 ubiquitin ligase COP1, with several transcription factors (Liang et al., 2019). For example the WRKY transcription factor, WRKY36, acts as a repressor upstream of HY5 (Yang et al., 2018) (**Figure 8**). WRKY36 is transcriptionally induced by UV-B in an UVR8 independent manner and its direct interaction with UVR8 depends on the presence of UVR8 in the same cellular compartment. Upon UV-B, UVR8 accumulates in the nucleus were it associates with WRKY36 and releases WRKY36 from the *HY5* promoter. The UVR8/WRKY36 interaction allows *HY5* transcription and consequently photomorphogenesis (Yang et al., 2018). It is important to note that WKRY36 interacts similar to COP1, with the C-terminus of UVR8 but not with COP1. Similar to *gcn2-1*, *CHS* is higher expressed in *wrky36* mutants already under white light conditions compared to wildtype. Yang et al. (2018) proposed that under white light conditions HY5 is not out-competed by WRKY36 on its own promoter resulting in a constitutive higher expression of *HY5* and consequently *CHS* (Yang et al., 2018). Nevertheless, UV-B specific *WRKY36* repression and *HY5* expression needs the presence of UVR8 and thus is different from the GCN2 pathway (**Figure 8**). There is still a need to clarify whether the presence of monomeric UVR8 in the nucleus is the only signal for the UV-B dependent transcriptionally upregulation of *WRKY36*. Another recently revealed novel UV-B pathway component was identified through the interaction of activated UVR8 with the brassinosteroid induced transcription factors BRI1-EMS-SUPPRESSOR1 (BES1/BRZ2) and its interaction partner BES1-interacting Myc-like1 (BIM1) (Liang et al., 2018). The interaction with UVR8 releases these two transcription factors from the promoters of brassinosteroid regulated genes and as a consequence growth and in particular cell expansion of the hypocotyls are inhibited (Liang et al., 2018). Since also this signaling pathway needs the presence of UVR8 it is unlikely to be involved in the activation of GCN2 upon UV-B (**Figure 8**).

Explanations are still elusive why different action spectra are necessary for UVR8 monomerization and UV-B induced *HY5* expression and whether UVR8 is the only UV-B photoreceptor or if additional factors are needed to modulate the action of UVR8 (Brown et al., 2009; Díaz-Ramos et al., 2018). UV-B responsive phenomena in mutants of *uvr8* and photobiological studies indicate that UV-B signaling might also be triggered by other pathways (Ulm et al., 2004; Safrany et al., 2008; Gardner et al., 2009; Shinkle et al., 2010; Leasure et al., 2011; Lang-Mladek et al., 2012; Tilbrook et al., 2013; Xie et al., 2015; O’Hara et al., 2019). Studies also suggested that reduced pterin may be a chromophore for a putative UV-B photoreceptor (Galland and Senger, 1988; Takeda et al., 2014). In mammals two major UV-B pathways have been proposed (Fritsche et al., 2007). One pathway is initiated due to the formation of pyrimidine dimers. The other pathway is independent of DNA damage and involves the cell surface arylhydrocarbon receptor (AhR) (Fritsche et al., 2007; Esser et al., 2013; Pollet et al., 2018). Upon UV-B, tryptophan forms an AhR ligand which upon binding triggers the translocation of AhR to the nucleus where detoxification genes are induced (Fritsche et al., 2007). In addition, UVB-activated AhR initiates endocytosis of the epidermal growth factor receptor (EGFR) and activates EGFR dependent phosphorylation of the mammalian MAP kinases, ERK1/2 (Fritsche et al., 2007).

From yeast to mammals GCN2 belongs to the integrated stress response pathway which is critical for adaptation. This pathway promotes cellular recovery upon stresses by balancing nutrient availability with protein translation and growth. In *Arabidopsis*, GCN2 is the only kinase, which phosphorylates eIF2α upon several stress conditions and, as it is shown in this work, also UV-B. The molecular mechanism of GCN2 activation has been intensively studied in yeast and mammalian cells. Accordingly, GCN2 binds uncharged tRNAs leading to a conformational change which exposes its kinase domain. GCN2 interacts also with ribosomes and a regulatory complex of GCN1 and the ATP-binding cassette protein GCN20 (Sattlegger and Hinnebusch, 2000; Sattlegger and Hinnebusch, 2005; Castilho et al., 2014). Recently it has been shown that mammalian GCN2 is even higher stimulated by ribosomes and purified ribosomal P-stalk complexes than deacylated tRNAs (Inglis et al., 2019). Thus one might speculate that UV-B induces structural changes on the ribosome which activate GCN2 at a posttranscriptional level. RNA-ribosomal protein crosslinks can be induced in maize within 2 h of filtered broad band UV-B (Casati and Walbot, 2004a). Thus we hypothesize that these crosslinks induce structural changes that either activate GCN2 through direct binding, or is responsible for the overaccumulation of uncharged tRNAs. Activated GCN2 phosphorylates eIF2α and thereby changes the population of translated mRNAs. In yeast and mammalian cells GCN2 inhibits global protein synthesis while allowing the translation of selected mRNAs. These mRNAs contain several short upstream open reading frames in the 5’ untranslated region reviewed in (Sonenberg and Hinnebusch, 2009; Pakos‐Zebrucka et al., 2016). Transcriptome and in particular proteome studies after UV-B exposure in maize have shown that genes coding for components of the translational machinery are overrepresented suggesting that ribosomes are newly synthesized for revival of translation (Casati and Walbot, 2003; Casati and Walbot, 2004b).

We propose following explanation for the phenotypes of *gcn2* mutants upon UV-B radiation. GCN2 is needed under stressful conditions and involved in the balancing of energy use. GCN2 regulates transcription only indirectly by for example supporting the preferential transcription of upstream open reading frame containing mRNAs. That the UV-B related *CHS* gene is higher expressed under control white light conditions indicate that GCN2 might have an impact on gene expression also under non-stressful conditions. GCN2 might be involved in the transcriptional repression of the energy demanding biosynthesis of stress protective metabolites such as components of the phenylpropanoid pathway. In *gcn2* mutants we hypothesize that these metabolites or other protective features would be not suppressed and thus *gcn2* mutants contain a constitutive higher protection against UV-B induced DNA damage. Therefore the DNA damage response gene *RAD51* is also less activated (**Figure 3E**) and CPD formation is reduced (**Figure 4**). Also the rate of translation is higher in *gcn2* mutants compared to wildtype and is less affected by UV-B (**Figure 6B**). The increased tolerance in relation to growth parameters and fecundity of *gcn2* mutants to a daily dose of elevated UV-B is probably the result of a combined action of all the three protection levels, i) constitutive transcription of a biosynthesis gene for potential UV-B scavenging components, ii) less CPD formation, and iii) continuation of higher rates of translation under UV-B.

## Data Availability Statement

All datasets for this study are included in the article/**Supplementary Material**.

## Author Contributions

JG and M-TH designed and supervised the project. PL did together with IF the eIF2α phosphorylation assays. PL did the gene expression and together with M-TH the growth experiments. KS-D, LZ, and JR performed the PU experiments and quantifications. JR did the CPD quantifications. M-TH, PL, LZ, JR, and JG analyzed the data. PL and M-TH wrote the draft. All authors discussed the results and commented on the manuscript.

## Funding

PL and KS-D were financed by ERASMUS fellowships. Further support came from the Austrian Science Fund grant F3707-B22 and I1725-B16 and the COST-Action UV4Growth.

## Conflict of Interest

The authors declare that the research was conducted in the absence of any commercial or financial relationships that could be construed as a potential conflict of interest.
