# Supplementary material for: Involvement of the eIF2α Kinase GCN2 in UV-B Responses
Source: Front Plant Sci. 2019 Nov 28;10:1492. doi: 10.3389/fpls.2019.01492 (PMC6892979; doi:10.3389/fpls.2019.01492)
Supplement: Supplementary file 1 [file DataSheet_1.pdf]

Involvement of the eIF2a kinase GCN2 in UV-B responses

Paula Llabata, Julia Richter, Isabel Faus, Karolina Słomińska-Durdasiak, Lukas Hubert Zeh, Jose Gadea, Marie-Theres Hauser

Supplemenatary Material

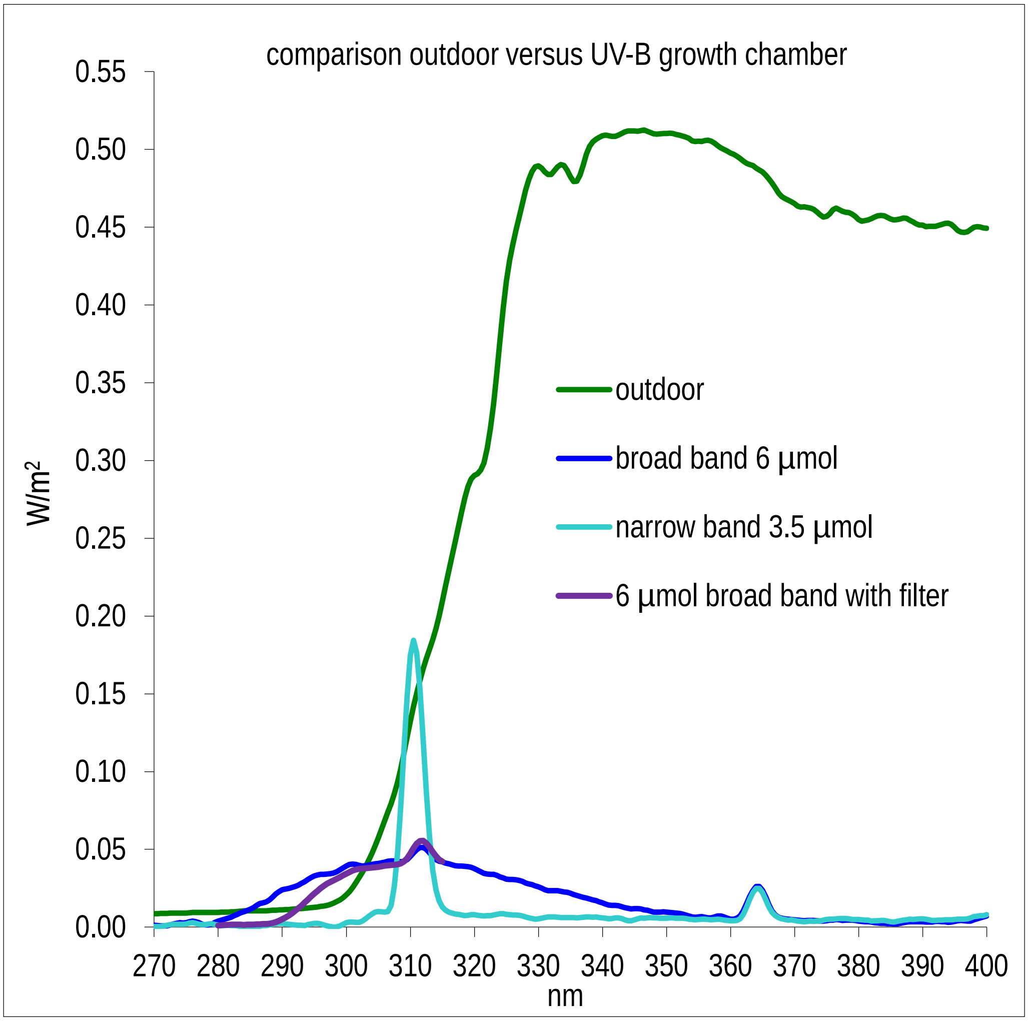

Supplementary Figure S1 Light spectra of the filtered and unfiltered broad and narrow band UV-B lamps in relation to a sun spectra taken at noon on a cloud less day in the middle of June in Vienna.

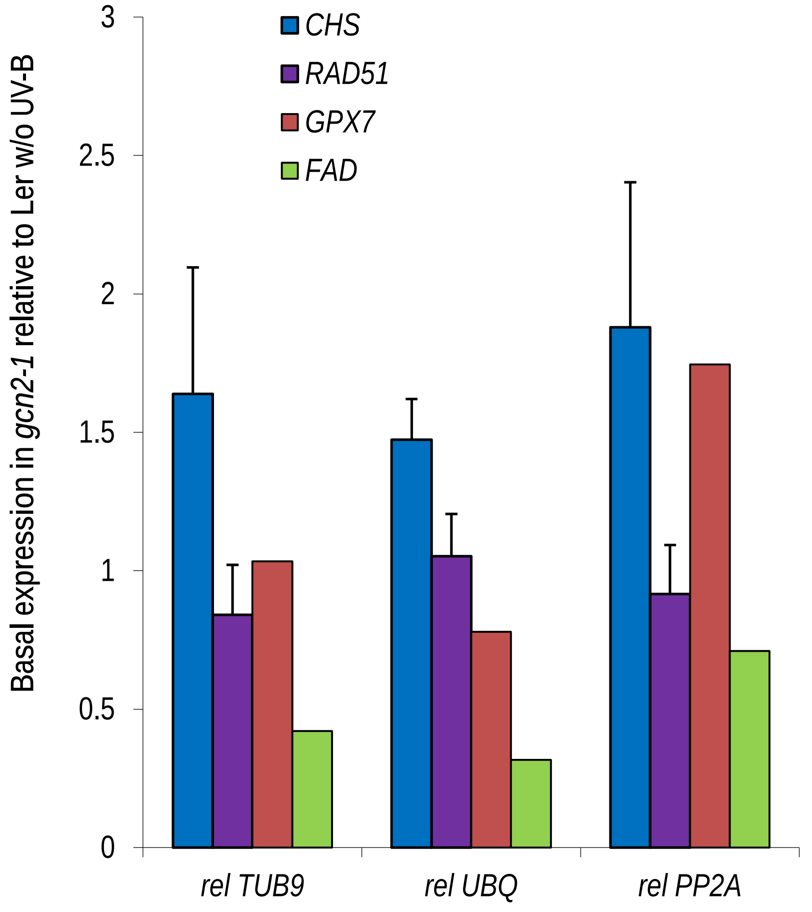

Supplementary Figure S2 Normalized expression with three reference genes, *TUB9*, *UBQ* and *PP2A*. *CHS* is constitutively upregulated in *gcn2-1*, while *FAD* is lower expressed in relation to the wildtype, Ler. *RAD51* is similarly expressed in *gcn2-1* and Ler independent of the reference genes used for normalization. For *GPX7* normalization with *TUB9* and *UBQ* would result in a similar expression to wildtype while with *PP2A* indicates a constitutive higher expression in *gcn2-1*. However if the normalization is done with all three reference genes, the difference is not significant (see Figure 3A.)

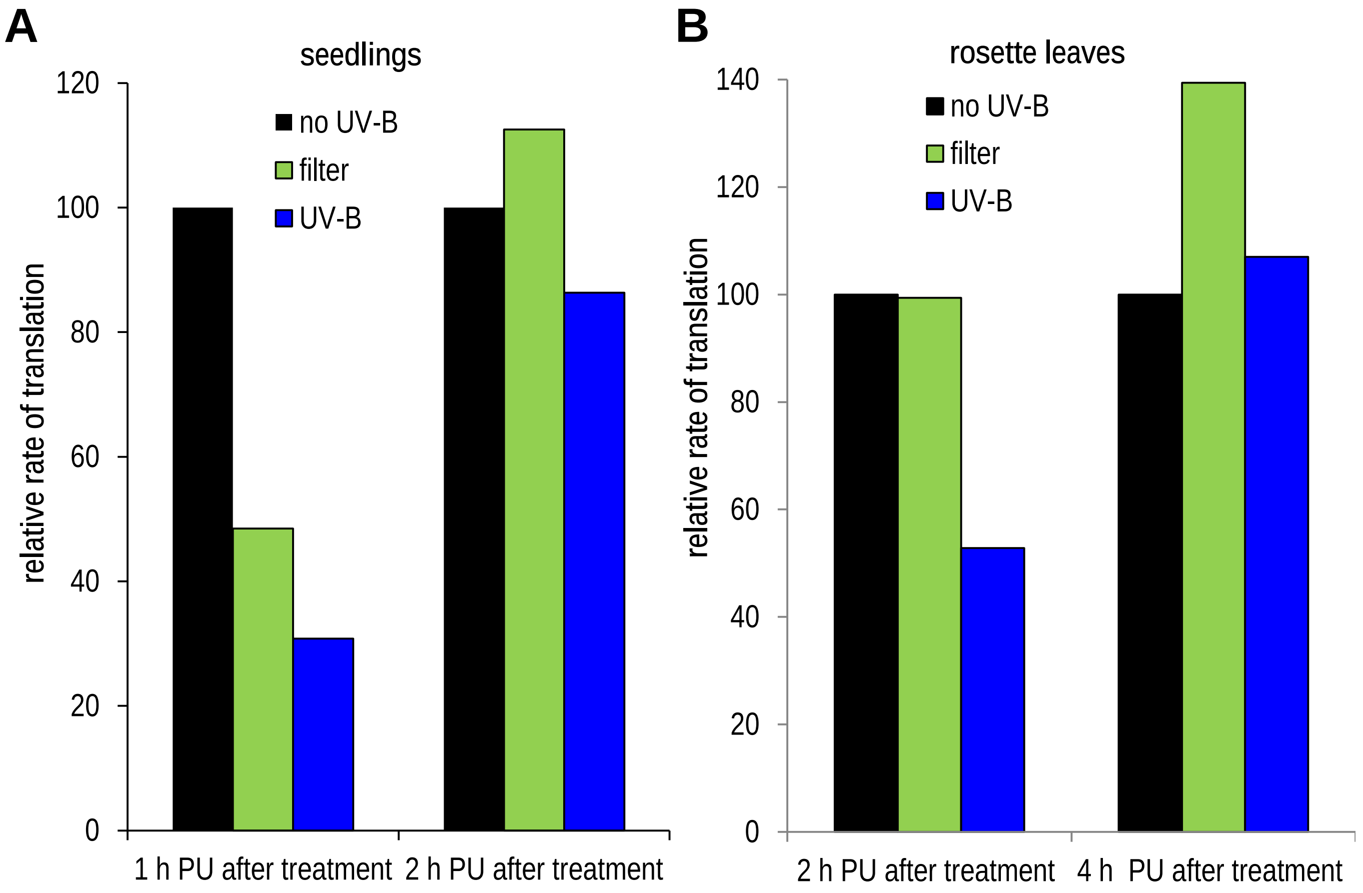

Supplementary Figure S3 Rate of translation at different time points after UV-B in **(A)** seedlings and **(B)** rosette leaves. Samples were treated for 1 h with either filtered or unfiltered broad band UV-B and PU was added either 1 h, 2 h or 4 h after UV-B shut down. **(A)** seedlings, **(B)** rosette leaves.

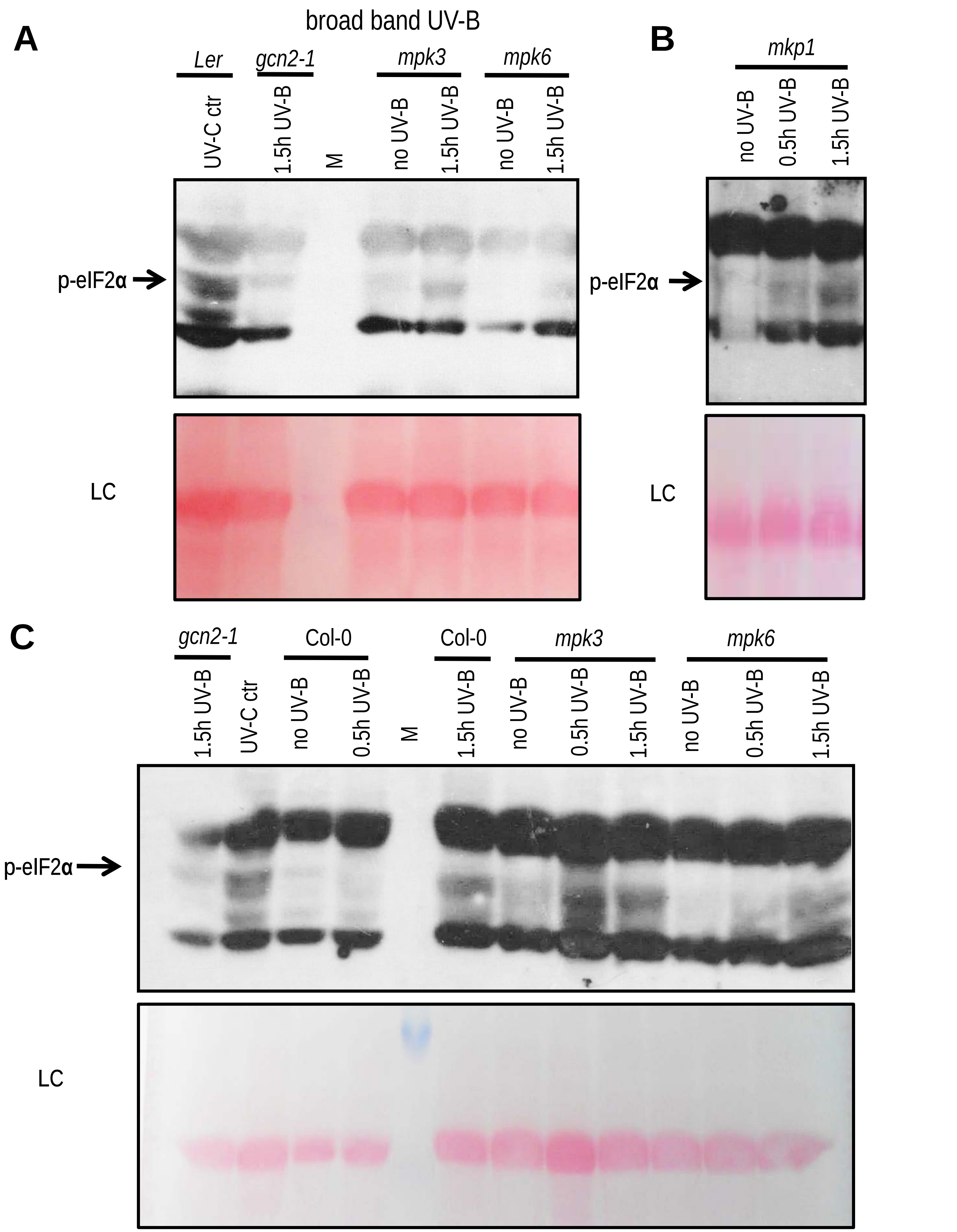

Supplementary Figure S4 Western blots assaying the activation of GCN2 via eIF2α phosphorylation in mutants of the MAP kinase stress signaling pathway. **(A)** Mutants and wildtype before and 1.5 h after the onset of 6 µmol m^{-2} s^{-1} broad band UV-B. **(B)** eIF2α phosphorylation in the mutant of the negative regulator of stress signaling *mkp1* after 0.5 h and 1.5 h of 10 µmol m^{-2} s^{-1} broad band UV-B. **(C)** Time course of mutants and wildtype before, 0.5 h and 1.5 h after the onset of 10 µmol m^{-2} s^{-1} broad band UV-B. Equal amount of protein (20 µg) was loaded on 10% SDS-PAGEs. LC: Loading control

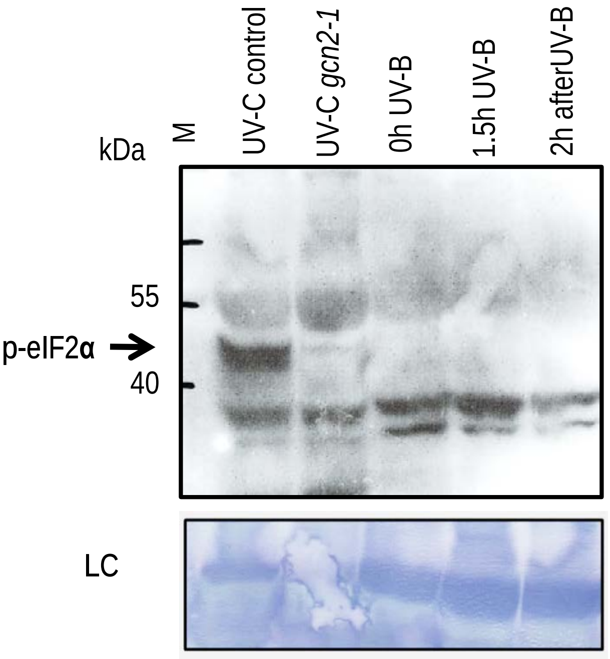

Supplementary Figure S5 Narrow band UV-B does not activate GCN2 assayed through Western blot analyses for eIF2α phosphorylation. Equal amount of protein (20 µg) was loaded on 10% SDS-PAGEs. After blotting the separated proteins, the membrane was probed for phosphorylated eIF2α. LC: Loading control

Supplementary Table S1: Primer characteristics for RT-qPCR

Primer Name	Primer Sequence 5'-3'	Fragment size in bp	Efficiencies
4g20890_TUB9-F	GTACCTTGAAGCTTGCTAATCCTA	cDNA 360, gDNA 470	0.77 +/-0.04
4g20890_TUB9-R	GTTCTGGACGTTCATCATCTGTTC		
1g13320_PP2A-F	TCTAGAGATGATTAACAACCCA	cDNA 401, gDNA 615	0.92 +/-0.06
1g13320_PP2A-R	ACAACACACGACAAAGTATCG		
3g62250_CUBQ	CTCCTTCTTTCTGGTAAACGT	gDNA = cDNA 426	0.74 +/-0.01
3g62250_NQ	AACCCTTGAGGTTGAATCATCC		
5g13930_CHS-F	ACTACTTCCGCATCACCAACA	gDNA = cDNA 197	0.72 +/-0.01
5g13930_CHS-R	GCTTAGGGACTTCGACCACCAC		
1g26380_ FADoxred-F	CGAAAAACACGAGGTTCTCG	gDNA = cDNA 290	0.71 +/-0.03
1g26380_ FADoxred-R	CCTCATCGATCTTCACGTAG		
4g31870_ GPX7- F	TGCAGCAGAGAAGTCTGTTC	gDNA 828, cDNA 369	0.88 +/-0.03
4g31870_ GPX7-R	ATCACCAAGGAAACCACCAG		
5g20850_RAD51-R	TGTTGTGGTGGCATGAGCCA	gDNA 318, cDNA 229	0.83 +/-0.04
5g20850_RAD51-F	ATAGTGCTACCGCTCTCTAC		
